# Correlation between μCT imaging, histology and functional capacity of the osteoarthritic knee in the rat model of osteoarthritis

**DOI:** 10.1186/s12967-015-0641-7

**Published:** 2015-08-25

**Authors:** Cedo M. Bagi, David E. Zakur, Edwin Berryman, Catharine J. Andresen, Dean Wilkie

**Affiliations:** Pfizer Global Research & Development, Global Science & Technology, 100 Eastern Point Road MS 8274-1359, Groton, CT 06340 USA

**Keywords:** Osteoarthritis, Articular cartilage, Subchondral bone, Dynamic weight bearing, μCT, EPIC μCT, Histology

## Abstract

**Background:**

To acquire the most meaningful understanding of human arthritis, it is essential to select the disease model and methodology translatable to human conditions. The primary objective of this study was to evaluate a number of analytic techniques and biomarkers for their ability to accurately gauge bone and cartilage morphology and metabolism in the medial meniscal tear (MMT) model of osteoarthritis (OA).

**Methods:**

MMT surgery was performed in rats to induce OA. A dynamic weight bearing system (DWB) system was deployed to evaluate the weight-bearing capacity of the front and hind legs in rats. At the end of a 10-week study cartilage pathology was evaluated by micro computed tomography (μCT), contrast enhanced μCT (EPIC μCT) imaging and traditional histology. Bone tissue was evaluated at the tibial metaphysis and epiphysis, including the subchondral bone. Histological techniques and dynamic histomorphometry were used to evaluate cartilage morphology and bone mineralization.

**Results:**

The study results showed a negative impact of MMT surgery on the weight-bearing capacity of the operated limb. Surgery caused severe and extensive deterioration of the articular cartilage at the medial tibial plateau, as evidenced by elevated CTX-II in serum, EPIC μCT and histology. Bone analysis by μCT showed thickening of the subchondral bone beneath the damaged cartilage, loss of cancellous bone at the metaphysis and active osteophyte formation.

**Conclusions:**

The study emphasizes the need for using various methodologies that complement each other to provide a comprehensive understanding of the pathophysiology of OA at the organ, tissue and cellular levels. Results from this study suggest that use of histology, μCT and EPIC μCT, and functional DWB tests provide powerful combination to fully assess the key aspects of OA and enhance data interpretation.

## Background

Osteoarthritis (OA) is a multifactorial disease characterized by gradual degeneration of the articular cartilage, associated with joint pain and dysfunction [1, 2]. The focal and progressive loss of hyaline articular cartilage in OA patients can be caused by natural aging, increased or non-physiological loads, obesity, trauma, hormonal disorders or a combination of several of these factors. Despite extensive research there is still no proven disease-modifying therapy and current treatments are palliative [3].

In rats, unilateral medial meniscal tearing (MMT) results in progressive deterioration and fibrillation of the articular cartilage, subchondral bone thickening and osteophyte formation [4–7]. Similar to human pathology, loss of joint matrix in the MMT rats is often most severe on the medial tibial plateau and at the outer half of the articular cartilage, whereas the inner half is less affected [7]. It was reported earlier that osteoarthritic rats, similar to human patients, tend to reduce weight bearing on the injured limb and shift their weight distribution to the contralateral limb [8–11]. Therefore, changes in the subchondral bone are believed to be caused by shifting of mechanical loads due to joint instability, but they can also result from the pain that accompanies surgery and/or the nerve compression and damage of the soft tissue and inflammation caused by altered biomechanics [12–14].

To acquire the most meaningful understanding of human OA, it is essential to select the disease model that closely resembles the human condition [15–17]. Ideally, the disease model and study design should closely reflect the pathological events in the articular cartilage, subchondral bone, and mechanical properties of human patients with OA. Accordingly, the methodology that is used to diagnose OA, monitor progression and assess the effect of therapy should be translatable to a clinical environment [18–20]. Nevertheless, researchers involved in preclinical studies using animals have several techniques at their disposal that cannot be replicated in the clinic. For example, zonal analysis of articular cartilage is the key histological method for the evaluation of cartilage damage in OA disease models [18], although histological techniques are not applicable in clinical situations. Recent improvements in non-destructive imaging modalities, such as magnetic resonance imaging (MRI) and μCT, have allowed for the creation of 3-dimensional (3D) images of bone and joints in laboratory animals and perhaps humans [21, 22]. Furthermore, use of μCT with a charged X-ray-absorbing contrast agent (EPIC μCT) has successfully delivered direct in situ visualization of the articular cartilage in preclinical OA models and in pilot studies in patients [23–26].

The primary objective of this study was to evaluate a number of analytic techniques and biomarkers for their ability to accurately gauge pathological change of cartilage and bone following the MMT surgery, and demonstrate consequence of cartilage deterioration on capacity of affected knee to withstand physiological loads. Demonstration of strong correlation between μCT imaging, histology, serum biomarkers and functional capacity of the OA knee will enable better assessment of disease modifying medicines under developed in animal models of OA, since new treatments will have to demonstrate not only capacity to heal or regenerate the cartilage and bone, but also to establish functional capacity of the diseased joint.

Animal models of OA have been associated with pain and discomfort to the animals, and scientists should make every effort to adhere to the principles of the “3Rs” when conducting their studies using these models [27, 28]. In this regard, the results of this validation of a MMT model of OA, as well as the methods, will allow scientists to apply appropriate models and methods in their studies and to generate reliable data to guide clinical trials, supporting the delivery of much-needed medicines to patients. Furthermore, it will justify the responsible use of animals in biomedical research.

## Methods

### Animals and management

Male, 4 months old Lewis rats (Charles River Laboratories, Portage, MI, USA) weighing roughly 350 g at the beginning of the experiments were used in this study. All the in vivo procedures were approved by the Institutional Animal Care and Use Committee (IACUC) and were performed in accordance with the Guide for the Care and Use of Laboratory Animals and with the US National Institutes of Health (NIH) Publication No. 85-23, revised 1996 [29]. The rats were pair housed in ventilated Innovive cages in a temperature- and humidity-controlled room on a regular 12 h light/dark cycle. Irradiated LabDiet™ 5053 (Purina, Richmond, IN, USA) and water were provided ad libitum. The animals were acclimated for 1 week prior to use in the 10-week study. Since all rats were of the same age (4 months) we randomization into study groups was based on their body weight the day before surgery. A group of 12 rats received sham surgery (Group 1), whereas 12 rats underwent MMT surgery (Group 2). Fluorochrome labels were administered to label actively mineralizing bone surfaces for analysis of new bone formation. Calcein (Sigma-Aldrich Cat# C-0875, St. Louis, MO, USA) was administered subcutaneously and intraperitoneally at 10 mg/kg on days 56 and 57 of the study. Alizarin (Sigma-Aldrich Cat# A-5533, St. Louis, MO, USA) was dosed intraperitoneally at 30 mg/kg on day 67 of the study.

### Surgery

The rats were induced and maintained under anesthesia using isoflurane. One dose of carprofen (Pfizer Animal Health, New York, NY, USA) and sustained-release buprenorphine (Zoopharm, Windsor, CO, USA) were administered prior to surgery for analgesic coverage. The right knee was shaved, aseptically prepared, and draped for surgery. In the sham group, a surgical approach to the medial collateral ligament on the right hind limb was completed, and the surgical incision was closed in 2 layers using absorbable sutures. In the surgery groups, medial meniscal tear (MMT) surgery was performed by transecting both the medial collateral ligament and medial meniscus of the right hind limb, followed by closure in 2 layers using absorbable sutures [7].

### Body weight sample collection and serum analyses

Body weight was recorded twice weekly throughout the study. At the end of the 10-week study the entire right hind limb was carefully harvested, skinned, and cleaned of the soft tissue, with care taken not to disrupt the knee joint. The limbs were wrapped in saline-soaked gauze and frozen at −20 °C for ex vivo imaging and histological analyses of the tibial articular cartilage and bone. Blood was collected at 5 and 10 weeks post-surgery by jugular venipuncture under isoflurane anesthesia. Serum was harvested and stored for serum chemistry and analyses of biomarkers of bone formation and bone and cartilage degradation. Osteocalcin was assessed by rat EIA kit (Cat# BT-490, Biomedical Technologies, Stoughton, MA, USA) and P1NP was quantified in serum samples by LC/MS method [30]. CTX was analyzed by rat LAPS™ Assay (Cat# AC-06F1), TRAP5b by Rat TRAP™ Assay (Cat# SB-TR102), and CTX II by CartiLaps™ Assay (Cat# AC08F1) all produced by Immunodiagnostics Systems (Scottsdale, AZ).

### Dynamic weight bearing

Dynamic weight-bearing (DWB) measurements were obtained before surgery, at week 5 and before euthanasia to assess the effects of surgery on the weight-bearing capacity of the hind and front legs. The DWB system (Bioseb, Boulogne, France) is a non-invasive method used to obtain the weight and surface area of all four feet in a freely moving animal [14, 31]. The data were analyzed using the DWB software, version 1.3. Zone parameters were set for the analysis as follows: ≥4 g for one sensor or a minimum of 3 adjacent sensors ≥2 g (to be considered a valid zone). For each time segment that was stable for more than 1 s, zones that meet the above criteria were validated and assigned as either right or left and front or rear. For each testing period, the animals were placed into the chamber and allowed 20–30 s to explore prior to data collection for a total of 3 min. Besides the body weight, the following parameters were measured: weight (g), percentage of weight (% weight) and surface area (mm [2] ) placed on the front left leg, front right leg, both front legs combined, rear left leg, rear right leg and both rear legs combined.

### Radiology

All the knee joints were X-rayed with the Faxitron Model MX20 specimen scanner (Faxitron Bioptics LLC., Tucson, AZ, USA) using the settings recommended by manufacturer; exposure time of 12–18 s at 31–35 kV. All the samples were imaged at 3× magnification and were positioned horizontally, with the center of the beam at the knee joint. Radiographic images were used to assess the gross anatomy of the region of interest to be evaluated by µCT and to inspect the bone samples for the presence of fractures or other bone abnormalities.

### μCT and EPIC μCT measurements

μCT was conducted on the right knee joint, utilizing a MicroCT 100^®^ computed tomography system (Scanco Medical, Bassersdorf, Switzerland) to obtain a scout 3D image of the knee and ensure that the samples were reproducibly scanned and analyzed exactly at the same region of interest (ROI) in each specimen and that the size of the ROI that we selected allowed for meaningful analysis of bone structures at the proximal tibial epiphysis and metaphysis.

Following imaging of the entire knee, the femur and tibia were carefully separated to ensure that the articular cartilage and meniscus of the joint were not disrupted. The tibia was then cut above the tibiofibular junction and the proximal tibia was then placed in a plastic custom-made positioning device to ensure consistent scanning. Pre-contrast scans of all the tibias were obtained using the MicroCT 100^®^ with the following parameters: 800 slices, a 10 µm resolution, a total scanned area of 8.0 mm [2], and source energy of 70 kVp, 115 µA at 8 W to capture the entire proximal tibia section.

Following pre-contrast μCT scans, 1.2 mL of Hexabrix (ioxaglate meglumine 39.3 % and ioxaglate sodium 19.6 %, Mallinckrodt, St. Louis, MO, USA) was added to a 15 mL conical tube and diluted with 1.8 mL of 0.1 M phosphate-buffered saline containing protease inhibitors (1 % Protease Inhibitor Cocktail Set I, CalBiochem, San Diego, CA, USA), yielding a 40 % solution of Hexabrix [23]. The tibia was then placed in this Hexabrix solution and was capped and incubated in a covered, rocking water bath at 37 °C for 3 h based on the quality of contrast scans obtained in the pilot study. After incubation period, the sample was removed, patted dry and placed in the plastic positioning device within a μCT holder, containing a small amount of saline to help maintain sample hydration during scanning with the MicroCT 100^®^. Post-soak scanning of the right tibia was performed in the same manner as described above, except that the parameters were set differently to better visualize the cartilage, with source energy of 55 kVp, 145 µA at 8 W and an average scan time of 42 min per sample [23].

### Proximal tibial metaphysis

The cancellous bone compartment of the metaphysis was analyzed 1 mm below the growth plate and extending 3 mm distally to include the metaphyseal secondary spongiosa. Cancellous bone was evaluated in a ROI drawn on 100 consecutive slices with a thickness of 1.0 mm that best represented the central segment of the tibia [32]. Cancellous bone parameters included bone mineral density, tissue volume (bone and bone marrow), bone volume, bone volume/tissue volume ratio, bone surface, bone surface/bone volume, trabecular number, trabecular thickness, trabecular separation, connectivity diameter, and structural model index.

### Subchondral bone (medial tibial plateau)

A 2.0 mm × 0.5 mm ROI was drawn on the pre-contrast images to include the cortical and cancellous subchondral bone underlying the articular cartilage. This region was drawn on the same 100 consecutive slices (1.0 mm total thickness) used for the metaphyseal and epiphyseal regions, which best represented the central segment of the tibia.

For zonal analysis of the subchondral bone at the medial tibial plateau, the lengths of tibial plateau for the sham and MMT rats were measured to average 2.4 and 3.0 mm, respectively. Longer plateau in MMT rats was due to formation of the osteophytes on the outside of the medial edge of the joint. Based on the pilot data, the medial tibial plateau was divided into 3 equal zones of 0.8 mm each starting from the inside of the plateau adjacent to the central collateral ligaments (Zone 3). Very edge of the plateau containing small part of the osteophytes were exclude from the analysis due to variation in size and shape, but also because excluded pieces of the plateau does not contain cartilage defect and therefore were not deemed relevant for evaluation of the subchondral bone and cartilage. Based on the pilot data and taking into account irregular geometry of the proximal tibia, we kept the depth (0.6 mm) and width (1.0 mm) of ROI similar for both sham and MMT rats since chosen size allows for reproducible measurements of the central cartilage and subchondral bone to always be captured and analyzed. Zone 1 (z1) was designated as the outside of the medial edge of the joint, Zone 2 (z2) was designated as the central zone, and Zone 3 (z3) was designated as the inside of the tibial plateau adjacent to the central collateral ligaments. The parameters of cortical and cancellous bone included bone volume and bone mineral density.

### Articular cartilage (medial tibial plateau)

Using the post-contrast scans, contour lines were drawn around a ROI that included the cartilage overlying the medial tibial plateau. The ROI was purposely drawn to include a small amount of bone and soft tissue around the cartilage to ensure that an appropriate threshold was selected to segment the cartilage from bone tissue and soft tissue according to histographic analysis of the tissues [18, 23, 24]. The lower threshold was determined to be 70, and the upper threshold was 400 (Gauss filter parameters: sigma = 1.2, support = 2). Contour lines were manually drawn with semi-automatic contouring applied every 3–10 slices over a total of 300 slices (3 mm) to capture most of the articular surface. The 3D morphology of the entire articular cartilage layer drawn was then visualized and quantified in terms of average cartilage thickness, volume and surface area using direct distance transformation algorithms [22–24, 33].

Other ROIs were drawn and analyzed on this central midpoint of the articular surface, corresponding to standard histological evaluation techniques for the articular cartilage [17]. The length of the medial articular cartilage was measured and divided into 3 zones of equal length as already described for the subchondral bone. The parameters of articular cartilage included cartilage volume (Car.V) and cartilage thickness (Car.Th).

### Histology

After the completion of EPIC μCT imaging of the articular cartilage, six tibias were randomly chosen and placed in 10 % neutral buffered formalin for 72 h prior to demineralization in Immunocal (Decal Chemical Corp. Tillman, NY, USA). The tibias were then processed into paraffin and were serially sectioned at ~200 µm intervals into 5 μm-thick sections for staining. The slides were stained with hematoxylin and eosin (H & E) and toluidine blue for general structural evaluation and with safranin O for the evaluation of cartilage damage. General cartilage degeneration included chondrocyte death/loss, proteoglycan loss, or fibrillation [7]. Thickness and degeneration of the articular cartilage at the medial tibial plateau were determined on three longitudinal sections of the proximal tibia using an ocular micrometer. Cartilage thickness was measured separately on each of three zones, as suggested in the literature [34, 35]. In addition, scoring of the osteophytes and categorization into small (up to 300 µm), medium (300–450 µm), and large (450–600 µm) was undertaken with the ocular micrometer. Marginal zone proliferative changes had to be ≥200 µm to be designated as osteophytes.

To assess active bone formation the remaining six tibias were embedded in methylmethacrylate and cut into three consecutive 8 μm-thick longitudinal sections using a polycut sliding microtome (Leica Biosystems, Nussloch, Germany) and one 20 μm-thick sections using a bone cutting and grinding system (Exakt Norderstedt, Germany). Unstained sections were used to assess new bone formation around osteophytes and at metaphysis.

### Statistical analysis

Data are reported as means ± standard deviations (SDs). Differences were tested for significance by Student’s *t* test for unpaired observations when comparing various parameters between the sham and MMT groups. Results were considered statistically significant if the *p* value was ≤0.05. The statistical analyses were performed using Sigma Plot software (version 12.2, Systat Software, Chicago, IL, USA).

## Results

### Body weight

All the study rats completed the study and exhibited 20 % weight gain during the course of the study, with no difference between the sham and MMT rats.

### Dynamic weight bearing

**Fig. 1:**
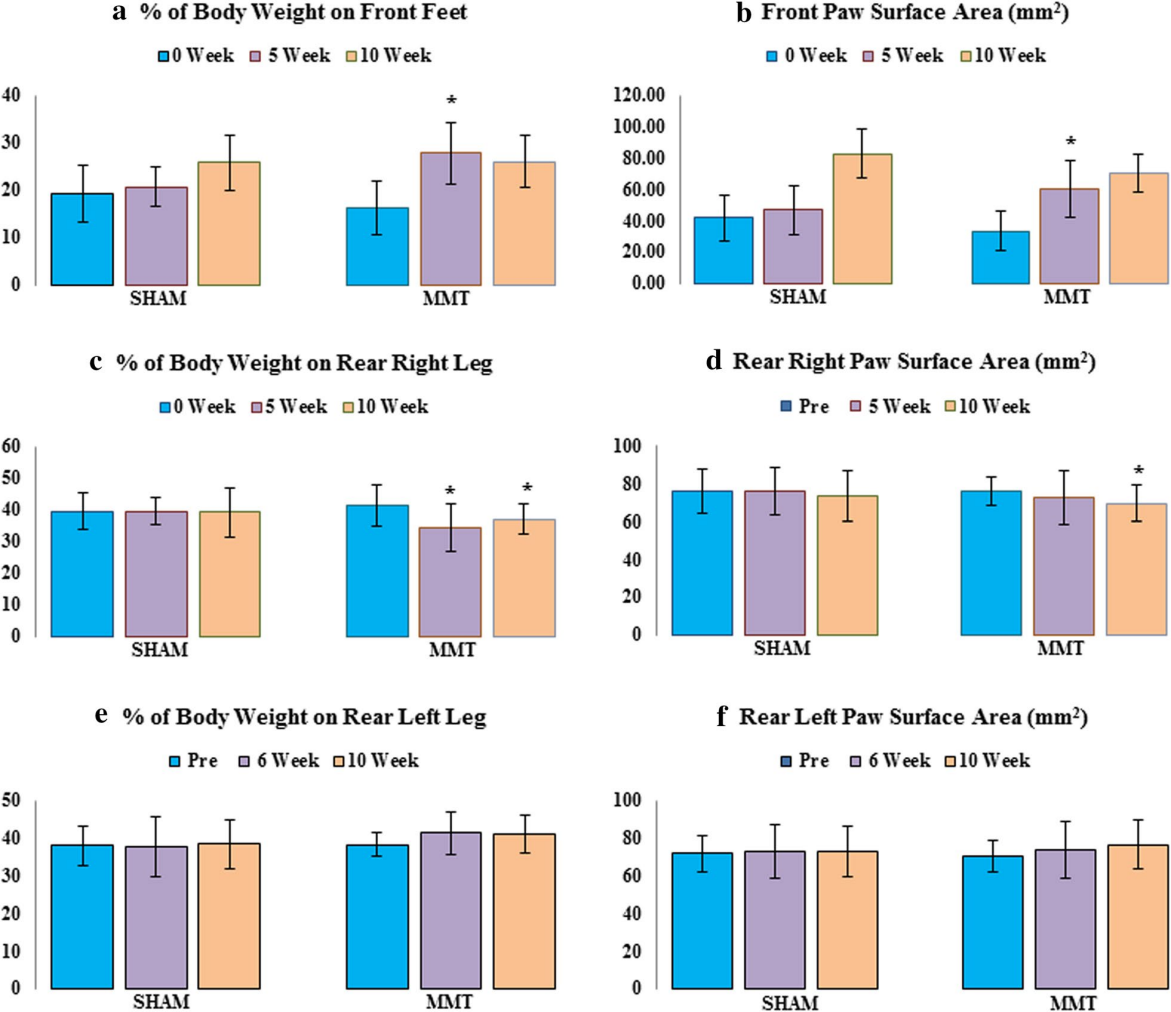
% change in body weight placed on the front legs (**a**), on the rear right leg (**c**), and on the rear left leg (**e**) as well as the paw surface area of the front legs (**b**), on the rear right leg (**d**) and on the rear left leg (**f**) in sham and MMT rats. *p < 0.05 relative to sham rats for the same time point

The MMT rats showed a different pattern of weight distribution compared to the sham rats. All the rats tended to shift weight bearing toward the front legs as they gained weight, but the MMT rats demonstrated this shift earlier; thus, at the 5-week time point, approximately 25 % of the body weight was already transferred to the front feet in MMT rats, compared to only 15 % in the sham rats. Additionally, the front paw-surface area was enlarged earlier in the MMT rats than in the sham rats. The total load placed on the hind legs was less in the MMT rats than in the sham controls, mainly because the rear right leg on which the MMT surgery was performed, exhibited less weight-bearing capacity and a smaller paw surface area than the right leg in the sham rats. Also, there is visible trend in MMT rats to increase the weight bearing of the left hind leg (Fig. 1).

### Serum chemistry and biomarkers

There were no significant differences in serum chemistry parameters between the sham and MMT rats. Analysis of terminal serum samples showed elevated CTXII in MMT rats compared to the shams (Table 1).

**Table 1:** Serum biomarkers of bone and cartilage metabolism at the end of the 1-week study

Parameter	Unit	SHAM	MMT
Osteocalcin	ng/mL	49.1 ± 13.1	38.1 ± 9.4
P1NP	ng/mL	146.0 ± 22.0	174.7 ± 26.1
CTXI	ng/mL	37.8 ± 4.9	40.5 ± 7.9
TRAP5b	ng/mL	3.2 ± 0.3	3.4 ± 0.9
CTXII	ng/mL	13.9 ± 3.6	19.2 ± 5.1*

* *p* < 0.05 relative to sham rats

### μCT evaluation of cancellous bone at the proximal tibial metaphysis

**Fig. 2:**
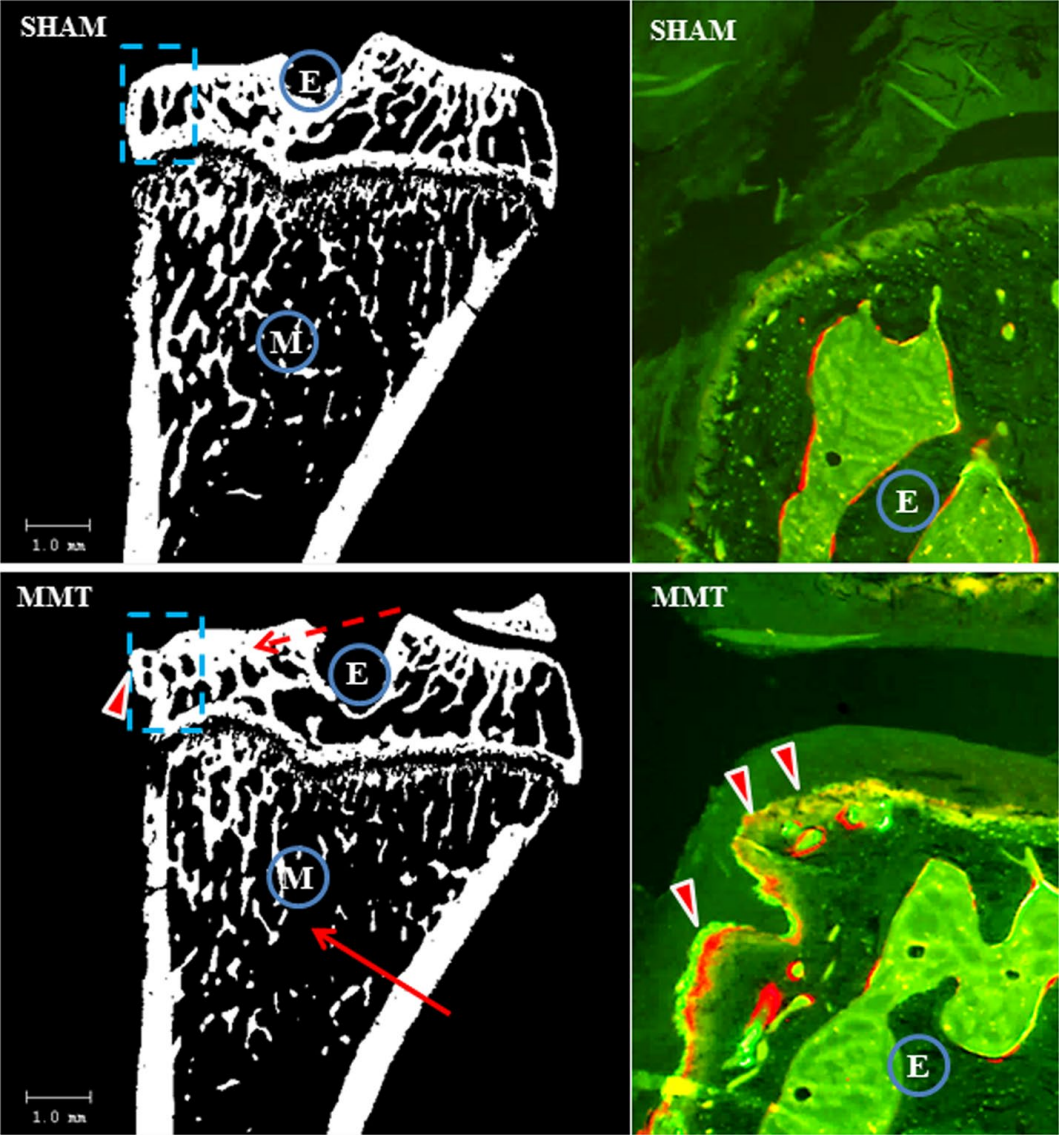
Two-dimensional µCT image of the proximal tibia from sham and MMT rat. Cortical and cancellous bone was measured for the entire epiphysis (*E*), whereas cancellous bone was evaluated at the metaphysis in the area of secondary spongiosa (*M*). *Solid arrow* indicates a lesser amount of cancellous bone in MMT rat; *dotted arrow* indicates thickening of the subchondral bone in MMT rat; *arrowhead* indicate osteophyte formation in MMT rat. *Dotted blue square* indicates area of epiphysis where UV micrographs are taken to show the new bone formation. *Arrowheads* indicate intensive bone remodeling of the osteophyte in MMT rat as judged by the presence of both calcein and alizarin red dye, whereas there is no osteophyte formation in the sham rat

The sham rats showed significantly higher BV (13 %), BV/TV ratio (18 %) bone surface (11 %), trabecular number (14 %) and connectivity diameter (21 %) and a lower trabecular separation index (24 %) compared to the MMT rats (Table 2; Fig. 2).

**Table 2:** mCT analysis of cancellous bone at the proximal tibial metaphysis

Parameter	Unit	SHAM	MMT
BMD	g/cm^2^	978.51 ± 10.95	985.89 ± 7.05
Tissue volume	mm^3^	11.63 ± 0.71	12.12 ± 0.75
Bone volume	mm^3^	2.01 ± 0.22	1.74 ± 0.23*
BV/TV	ratio	0.17 ± 0.02	0.14 ± 0.02*
Bone surface	mm^2^	65.83 ± 6.44	58.75 ± 8.05*
BS/BV	ratio	32.79 ± 1.85	33.87 ± 1.49
Trabecular number	1/mm	2.83 ± 0.24	2.42 ± 0.25*
Trabecular thickness	μm	0.061 ± 0.003	0.059 ± 0.003
Trabecular separation	μm	0.29 ± 0.03	0.36 ± 0.05*
Connectivity diameter	1/mm^3^	52.33 ± 7.58	41.17 ± 6.10*
SMI	1	2.04 ± 0.12	2.15 ± 0.10

* *p* < 0.05 relative to sham rats

### μCT evaluation of subchondral bone and cartilage at the medial tibial plateau

**Fig. 3:**
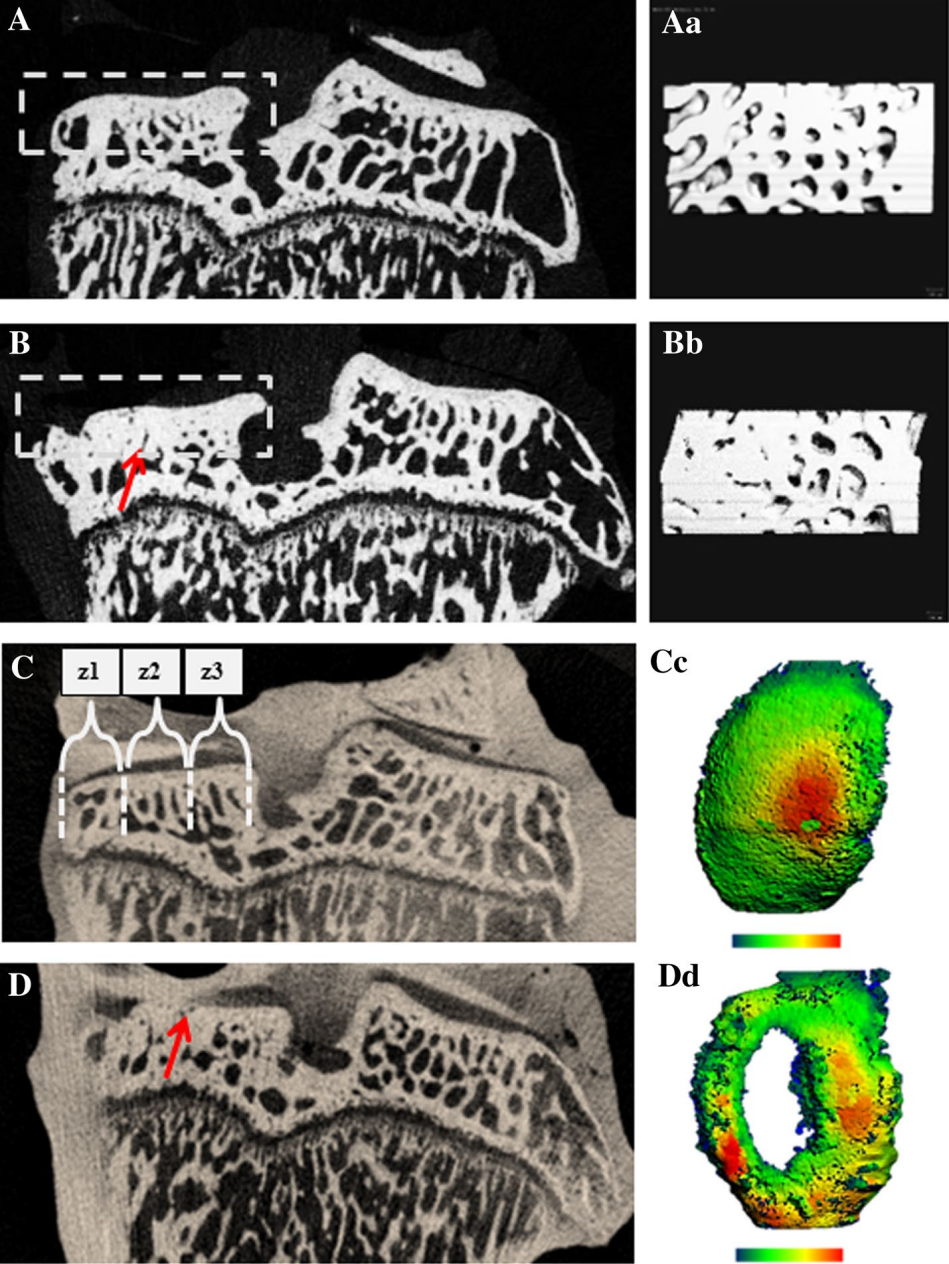
2D (**a**, **b**) µCT images of the tibial epiphysis from a sham rat (**A**) and MMT rat (**B**). *Doted squares* indicate area of subchondral bone used for zonal analysis. 3D images of subchondral bone are depicted for sham (**Aa**) and MMT rat (**Bb**). *Arrow* indicates thicker subchondral bone in MMT rat. **C** (sham) and **D** (MMT) shows a contrast µCT image of the proximal tibial epiphysis of the same rats shown in **A** and **B**. The length of the medial tibial plateau was measured for each sample and was then divided into three zones ranging from 0.8 to 1.0 mm in length. Zone 1 (*z1*) was placed on the outside of the medial edge of the joint, and Zone 3 (*z3*) was placed on the inside of the tibial plateau, adjacent to the central collateral ligaments as exemplified in **C**. Zones are delineated by *dotted lines*. **Cc** (sham) and **Dd** (MMT) show color thickness (“heat”) maps of the articular cartilage at the medial tibial plateau. *Arrow* indicates a lack of viable cartilage that covers the medial tibial plateau in MMT rat

Although the subchondral bone volume was larger in the MMT rats than in the sham controls in all 3 zones, the most significant difference was measured in Zones 1 and 2. There was no difference in BMD between the sham and MMT rats. Cartilage volume was greater in Zones 1 and 3 but was significantly less in Zone 2 in the MMT rats, compared to the sham controls. Cartilage thickness was greater in Zone 1 but was significantly less in Zone 2 (*p* < 0.001) in the MMT rats, compared to the sham controls. Cartilage thickness in Zone 3 was somewhat higher in sham rats relative to MMT rats (Table 3; Fig. 3).

**Table 3:** Zonal analysis of subchondral bone and articular cartilage covering the medial tibial plateau

Parameter	Unit	Zone	SHAM	MMT	% difference
Bone volume	mm^3^	z1	0.37 ± 0.03	0.46 ± 0.05**	24.4↑
		z2	0.42 ± 0.02	0.49 ± 0.04**	16.7↑
		z3	0.45 ± 0.04	0.47 ± 0.04	4.4↑
		z1	1003.79 ± 14.52	980.10 ± 16.80	2.4↔
Bone mineral density	g/cm^2^	z2	1020.19 ± 17.80	1031.94 ± 7.56	1.1↔
		z3	984.21 ± 24.53	978.70 ± 11.51	0.6↔
		z1	0.12 ± 0.02	0.19 ± 0.06*	58.3↑
Cartilage volume	mm^3^	z2	0.20 ± 0.02	0.11 ± 0.04**	45.0↓
		z3	0.19 ± 0.04	0.23 ± 0.04*	21.1↑
		z1	0.14 ± 0.02	0.18 ± 0.06*	28.6↑
Cartilage thickness	mm	z2	0.23 ± 0.02	0.15 ± 0.04**	34.8↓
		z3	0.23 ± 0.04	0.20 ± 0.03	12.1↓
Osteophyte		z1	None	Large	

The subchondral bone and articular cartilage were divided into 3 zones; Zone 1 (z1) was placed on the outside of the medial edge of the joint, and Zone 3 (z3) was placed on the inside (central) of the tibial plateau, adjacent to the central collateral ligaments. There were also large osteophytes present in Zone 1 in the MMT rats

* *p* < 0.05 or ** *p* < 0.001 relative to sham rats

### Bone dynamic

Dynamic histology revealed active bone remodeling and active osteophyte formation at the medial epiphysis of the MMT rats compared to the sham rats, whereas at the tibial metaphysis, the sham rats exhibited more bone formation and more cancellous bone than the MMT rats (Fig. 2).

### Histology

**Fig. 4:**
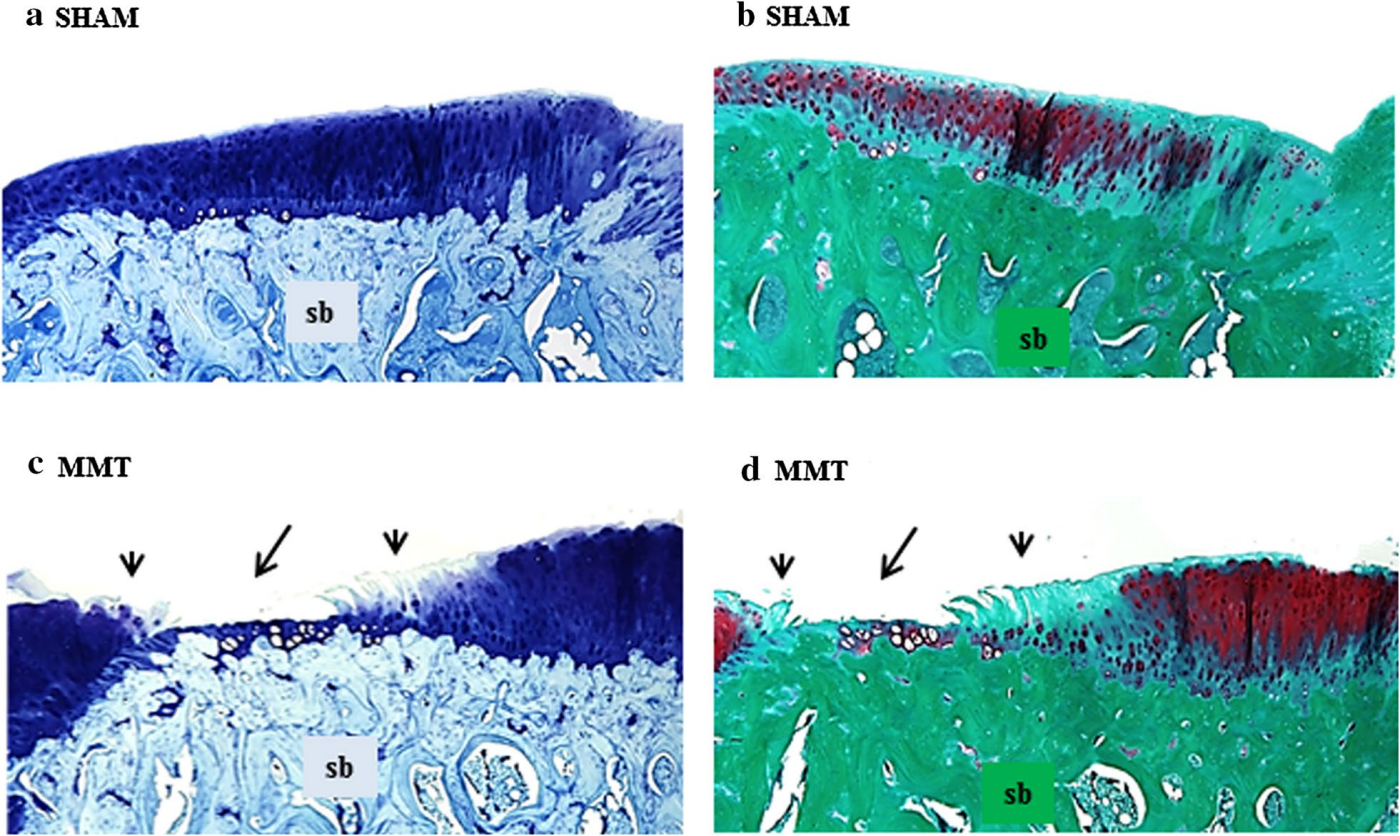
Typical view of the articular cartilage at the medial tibial plateau from sham and MMT rats stained with toluidine blue (**a**, **c**) and safranin O (**b**, **d**). *Arrows* indicates degradation of the articular cartilages in Zone 1 and 2, and *arrowheads* indicate cartilage fibrillation in Zones 1 and 3

Histological evaluation of articular cartilage revealed classic images of cartilage degradation caused by the MMT surgery: thinning to complete absence of the cartilage due to chondrocyte death or atrophy, cartilage fibrillation and the presence of osteophytes. In the control rats, the articular cartilage at the medial tibial plateau grew progressively thicker from the most medial (Z1) to the most lateral part (Z3), as depicted on by morphometry on histological sections (Table 4; Fig. 4). MMT surgery triggered thickening of the cartilage in the most medial half of Zone 1 next to the osteophytes. The cartilage was thin and occasionally completely missing in Zone 2 in the MMT rats. Cartilage thickness in Zone 3 in MMT rats was decreased relative to sham controls, but the change was not as drastic as in the Zones 1 and 3 (Table 4; Fig. 4). Damage score, Cartilage matrix loss width for 0, 50 and 100 %, Total cartilage degeneration width and Significant cartilage degeneration width were all significantly (*p* < 0.001) elevated in MMT rats compared to sham controls (Table 4).

**Table 4:** Zonal measurement of cartilage thickness and measurement and scoring of the osteophytes on histology slides (safranin O) at the medial tibial plateau

Parameter	Unit	Area	SHAM	MMT
Cartilage thickness	μm	z1	536.15 ± 18.78	1035.51 ± 34.62**
		z2	916.96 ± 21.14	182.76 ± 20.05**
		z3	1092.5 ± 23.13	1016.00 ± 25.81
Damage score (1–5)	score	z1–z3	0.2 ± 0.04	4.3 ± 1.62**
Cartilage matrix loss width	μm	0 %	307.44 ± 145.73	4586.24 ± 846.11**
		50 %	126.53 ± 82.22	2631.76 ± 350.87**
		100 %	46.36 ± 145.73	1843.15 ± 304.20**
Total cartilage degeneration width	μm	z1–z3	312.46 ± 35.26	4965.55 ± 930.87**
Significant cartilage degeneration width	μm	z1–z3	6.32 ± 10.50	1987.53 ± 962.81**
Osteophyte size	μm	z1	26.42 ± 15.81	480.19 ± 62.33** (large)

* *p* < 0.05 or ** *p* < 0.001 relative to sham rats

## Discussion

Similar to knee OA in humans, meniscal injury in rats results in degenerative changes of the articular cartilage, leading to chondrocyte degeneration and death, loss of proteoglycans and matrix fibrillation, with subsequent changes in subchondral bone and osteophyte formation. Progressive deterioration of the articular cartilage compromises joint stability and mechanical loading on the knee, causing disabilities associated with intractable pain [5–7]. This study was undertaken to assess the usefulness of the DWB system to assess biomechanical consequence of cartilage and bone pathology in OA rats and to correlate serum biomarkers of cartilage and bone metabolism with μCT and histology data. Establishing solid correlation between joint function, serum biomarkers, histology and μCT imaging will help to monitor disease progression and treatment outcomes in preclinical studies, but also in the clinic where histology methods are not applicable.

Surgical procedures provoked a transient decrease in body weight that was most likely due to stress and postoperative pain because there was no evidence of the excessive postoperative swelling that typically indicates joint infection. The cartilage damage in the MMT rats was highly consistent between animals, as judged by contrast μCT and histology, and it had an impact on the ability of the damaged knee to withstand weight-bearing loads. The total weight bearing imposed on the rear and front legs in all the rats increased over time as the rats became heavier. Increase in body weight shifted weight distribution toward the front legs in all the study animals. However, the MMT rats shifted their body weight toward the front legs earlier than the sham rats; therefore, at the 5-week time point, approximately 25 % of the body weight was already transferred to the front feet in the MMT rats, compared to only 15 % in the sham rats. In addition, the weight-bearing load placed on the right hind leg was smaller in the MMT rats than in the sham controls, because the rear right leg, on which the MMT surgery was performed, showed less weight-bearing capacity compared to the right leg in the sham rats. While other researchers have previously shown shift of weight distribution in osteoarthritic rats to the contralateral limb, they failed to notice the weight- shift toward the front paws [10, 11, 36, 37]. Patients with knee OA also show gait visible asymmetries through reductions in the stance time and peak vertical force of the OA-affected limb, while the same time overloading the contralateral knee [8, 9, 38]. Our data indicated that in contrast to bipeds, in which only option is to shift the weight to the contralateral limb, rats (quadrupeds) tend to alleviate mechanical imbalances associated with pain by shifting at least part of the weight burden onto their front legs, rather than simply overloading their contralateral limbs. In both humans and rats, mechanical loading drives changes in skeletal remodeling to adjust the bone mass and architecture to meet mechanical demands [12, 39–43]. It has been well established that in rats, cancellous bone at the tibial metaphysis rapidly responds to changes in mechanical loading due to the high bone turnover rate at this skeletal site [44, 45]. In our study, the reduced weight bearing activity of the right hind leg in MMT rats resulted in decreased structural parameters of the cancellous bone at the proximal tibial metaphysis.

In contrast to bone loss at the metaphysis, subchondral bone beneath the medial tibial plateau in the MMT rats was thicker, as demonstrated by μCT and confirmed by histology. Again, changes in local mechanical loads caused by deterioration of the articular cartilage triggered increased bone formation beneath the damaged cartilage to accommodate new mechanical needs. In addition, mechanical imbalance resulted in osteophyte formation, most likely as an adaptation of the skeleton to stabilize inured joints and to prevent further deterioration of the cartilage and bone while maintaining joint functionality [46–48]. Dynamic histomorphometry data showed that even after 10 weeks post-surgery, there was still active bone remodeling of the osteophytes in the MMT rats. Osteophytes can continue to limit joint movement and can be a source of joint pain, thus affecting bone and cartilage metabolism. Data collected by various methods have emphasized the importance of local biomechanics, showing that changes in weight-bearing loads within the same bone (tibia) can either increase resorption (metaphysis) or increase formation (subchondral bone, osteophytes) to adjust the bone mass and structure to new mechanical needs.

It was previously demonstrated that EPIC μCT imaging provided a reproducible method to assess 3-D distribution of glycosaminoglycans (GAGs) in the articular cartilage of laboratory rats [23, 49], and that imaging data strongly correlated with histological findings [21–23]. Three-dimensional images could be used to guide the histological evaluation of articular cartilage by indicating areas of cartilage damage so that areas warrant evaluation will not be missed on histological sections. Three-dimensional cartilage analyses by μCT unequivocally demonstrated the extent of cartilage damage following MMT surgery and micro-anatomical localization of the damage (Z1 and Z2), as well as adaptation and thickening of the articular cartilage at the most medial corner of Zone 1, bordering the region of osteophyte formation. Imaging data of the articular cartilage were correlated well with traditional histological methods, as well as with serum biomarkers of cartilage degradation (CTX-II). Serum biomarkers of bone metabolism reflect the change in bone formation and bone resorption in the entire skeleton. We hypothesize that the magnitude of bone remodeling at the metaphyseal and subchondral bone (including ostephytes) was probably too small to be picked in the serum at the end of 10-week study. Also, serum biomarkers reflect only bone resorption and formation at the time of serum collection and this is probably another reason why these biomarkers have only limited diagnostic and prognostic value in OA patients [19, 50].

Use of EPIC μCT imaging, in combination with standard μCT and histological techniques, provided a powerful tool for detailed analysis of cartilage and bone because these two methodologies complemented each other by addressing changes at the organ, tissue and cellular levels. Histology and EPIC μCT imaging were successfully deployed in the past to advance development of novel therapies [6, 51]. Use of diverse technologies, such as the DWB system and dynamic histomorphometry, in preclinical studies is encouraged because these methods can shed light on different aspects of complex diseases and could enhance our understanding of disease pathology, thereby supporting the development of much-needed therapies. Collection of comprehensive data will also justify the use of laboratory animals in biomedical research to answer questions that cannot be addressed using ex vivo alternatives.

Implementation of multiple methods could be challenging on several levels including high cost and requirement of highly trained personnel, but also availability of sophisticated technologies. On the technical level, the biggest challenge in this study was to perform μCT, contrast enhanced EPIC μCT and histology measurements on the same tibia, mainly because of 3-h incubation time using hexabrix. Some of technical challenges can be resolved in the future since others have shown that shorter incubation time can be used without compromising data quality [52].

Collectively, the data from this study showed that even though histology, μCT and DWB methods could be used as stand-alone techniques, the combination of all three methods adds value as each method address different aspect of a complex disease. Although, some of the methods used in this study have limited application in clinic, their use in preclinical studies is warranted in order to enable correct interpretation of the data and better guide clinical research.

## Conclusions

Evaluation of a complex disease like OA requires use of diverse methods in order to fully address all aspects of the disease. In that regard, selection of the animal model and methods to evaluate cartilage and bone pathology and joint functionality is of the outmost importance. Since all currently used methods have limitations, deployment of several complementary methods is necessary to provide accurate interpretation of the data. Results from this study suggest that use of histology, μCT and EPIC μCT, and functional DWB tests provide powerful combination to fully assess the key aspects of OA and enhance data interpretation.
